# Left Ventricular Myxoma Leading to Stroke: A Rare Case Report

**DOI:** 10.1097/MD.0000000000001913

**Published:** 2015-11-13

**Authors:** Yan Kong, Huan Li, Jin Wang, Yongna Chai, Wuhui Hou, Ning Zhang

**Affiliations:** From the Department of Medicine Oncology, The Fourth Affiliated Hospital of Hebei Medical University, Shijiazhuang, Hebei (YK); Postgraduate School, Tianjin Medical University, Heping, Tianjin (HL); Department of Orthopedics, The Fourth Affiliated Hospital of Hebei Medical University, (JW); Department of Respiration, The Second Affiliated Hospital of Hebei Medical University (YC); Department of Cardiac Surgery (WH); and Department of Cardiology, The Fourth Affiliated Hospital of Hebei Medical University, Shijiazhuang, Hebei, P.R. China (NZ).

## Abstract

Primary cardiac tumors are rare, and most are myxomas. Only approximately 5% of cardiac myxomas originate from the ventricles.

We report the case of a 23-year-old man presenting with right hemiplegia and muscle strength degeneration under a diagnosis of stroke. Transthoracic echocardiography revealed a 29 × 26 mm mass arising from the anterior interventricular septum. The tumor was surgically removed, and histology confirmed the diagnosis of left ventricular myxoma.

We report its clinical features and treatment to add to the current knowledge.

## INTRODUCTION

Cardiac tumors are rare, the majority of which are myxomas, accounting for only 0.02% of primary tumors,^1^ and 75% of them are benign. Ventricular myxoma only accounts for approximately 5% of myxomas.^2^ Myxomas develop in all age groups. Patients range from stillborn infants to a 95-year-old woman, with an average age of 24 years.^1^ Myxomas are found particularly frequently between the 3rd and 6th decades of life.^3^ But there appears to be an increased incidence of right ventricular (RV) myxomas in children compared to that in adults.^4^ Recently, an increasing number of patients were diagnosed in routine examination before any symptom occurred. After the diagnosis is established, surgery should be performed as soon as possible to avoid severe complications or sudden death. Most cases obtained complete recovery after tumor resection. Recurrence happens in 1% to 3% of sporadic cases of cardiac myxomas, but the risk increases to 12% and 22%, respectively, for patients with familial and Carney complex myxomas.^5^ We herein report a case of an unusual left ventricular (LV) myxoma with embolization to the cerebral lead to stroke. The patient received tumor resection soon after the diagnosis was confirmed.

## CASE REPORT

**FIGURE 1:**
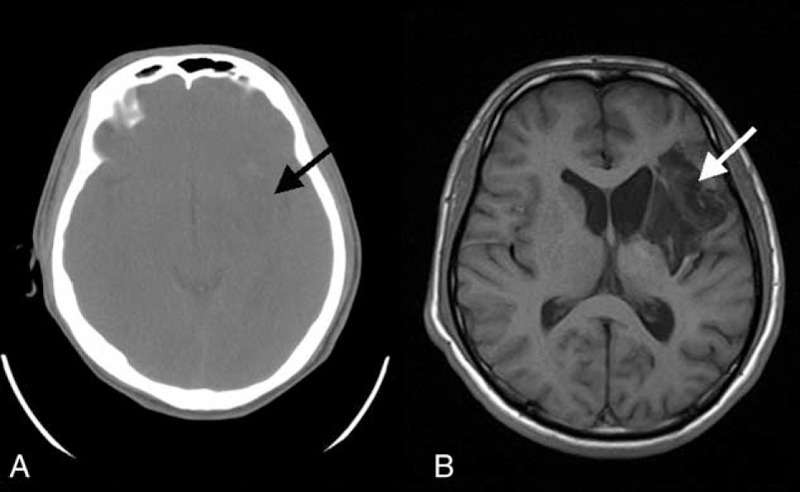
Brain CT (A) and MRI (B) showing a large infarct area (arrow) in the left basal ganglia, corona radiate, and temporal lobe. CT = computed tomography, MRI = magnetic resonance imaging.

**FIGURE 2:**
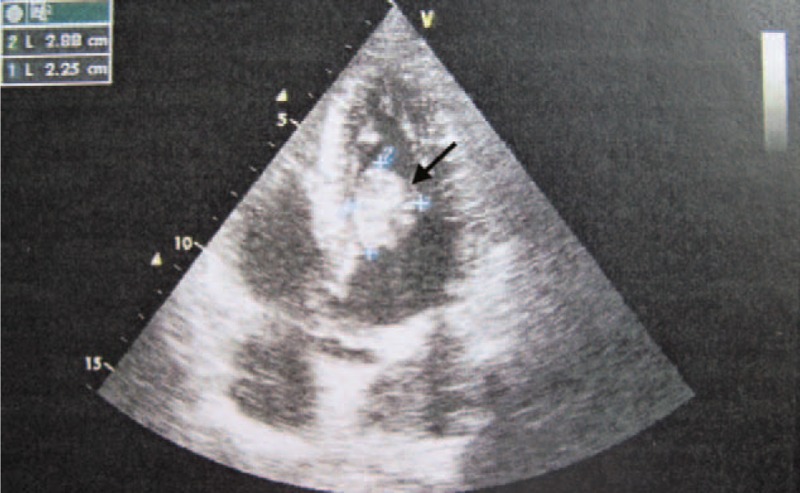
Transthoracic echocardiography showing a pedunculated heterogeneous mass (arrow) measuring 29 × 26 mm in the left ventricle.

**FIGURE 3:**
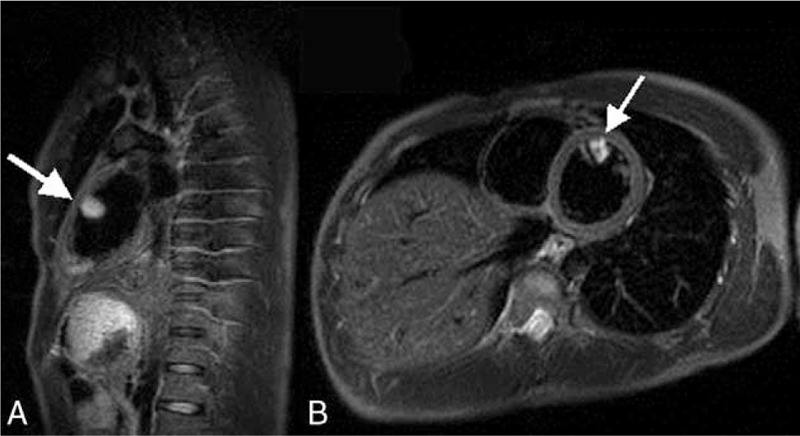
T1-weighted cardiac MR images showing that the mass (arrow) is attached to the anterior interventricular septum and anterior free wall of the left ventricle. MR = magnetic resonance.

A 23-year-old man was admitted to our hospital on June 16, 2014. He had a history of right hemiplegia for 12 hours with no apparent cause. He showed no cough, palpitations, breathlessness, chest pain, syncope, or loss of vision. He had no relevant family history, but had a history of 5 years smoking approximately 20 cigarettes per day. Physical examination revealed grade III muscle strength. Brain computed tomography (Fig. 1A) and magnetic resonance imaging (MRI) (Fig. 1B) showed a large infarct area in the left basal ganglia, corona radiate, and temporal lobe. Electrocardiography showed sinus rhythm with a heart rate of 78 bpm. Routine blood and urine examination revealed no abnormalities. Transthoracic echocardiography (TTE) revealed a pedunculated mass measuring 29 × 26 mm, arising from the anterior interventricular septum and anterior free wall of the left ventricle (Fig. 2), which was confirmed by cardiac magnetic resonance (Fig. 3). Therefore, ventricular myxoma was strongly suspected.

**FIGURE 4:**
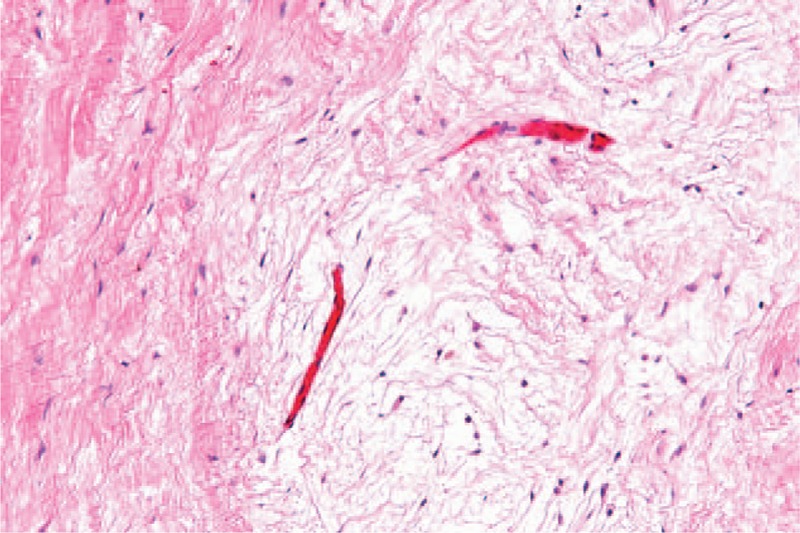
Histopathologic examination of the excised mass showing spindle-shaped mesenchymal cells in myxomatousstroma (HE = hematoxylin-eosin staining 100×).

After 3 months of conservative treatment, surgery for tumor resection was scheduled on September 26, 2014. Under general anesthesia and cardiopulmonary bypass, the patient underwent median sternotomy, and the tumor was excised completely. Postoperative histopathological examination was consistent with the diagnosis of myxoma (Fig. 4). The patient recovered uneventfully during 7 months of follow-up. His neurological symptoms improved gradually under conventional therapy including thrombolysis and anticoagulation.

The patient provided written informed consent for the publication of these case details and the consent procedure was approved by the Human Ethics and Research Ethics committees of the Fourth Hospital of Hebei Medical University.

## DISCUSSION

Cardiac myxoma is associated with embolism, obstruction to the outflow tract, arrhythmia, and other constitutional symptoms. Embolism occurs in 30% to 40% of all cases. The risk of embolization mainly depends on the morphology of myxoma rather than its size. There are 2 types of cardiac myxomas: friable polypoid type and smooth-surface rounded type. The polypoidal type tends to result in embolism because of its friable consistency and intracavitary location.^6^ Furthermore, the left ventricle has a higher risk of embolization since the high mobility and pressure in it. In the literature reviewed by Meller et al,^7^ 64% of the patients with LV myxoma presented with systemic embolization, most of which were cerebral. Other reported sites of embolism include the coronary artery, pulmonary artery, renal artery, and common femoral artery.

Atrial myxomas usually originate in fossa ovalis or its rim. In contrast, ventricular myxomas can arise from any portion of the ventricular chamber and may have either a broad base or a multicentric origin, which makes their obstructive symptoms various. RV myxoma causes pulmonary outflow tract obstruction, which leads to shortness of breath, edema, jugular venous distension, and other symptoms of congestive heart failure. Syncope, palpitations, and exertional chest pain are more commonly seen in LV myxoma.

Arrhythmia, mostly atrial fibrillation, occurs when cardiac conducting system is involved, which presents tight association with heart failure. Middlekauf et al^8^ reviewed 390 patients with severe heart failure and found 19% patients have a history of atrial fibrillation.

Cardiac myxomas produce interleukin (IL)-6, leading to constitutional manifestations such as recurrent fever, anemia, arthralgia, and weight loss. These nonspecific symptoms mostly arise from the elevation of IL-6 and disappear after removal of the tumor.

In conclusion, we report an unusual case of LV myxoma leading to stroke. Early detection such as TTE and cardiovascular magnetic resonance (CMR) can be used and patients could acquire complete recovery after total tumor resection.
